# Efficacy and safety of thrombectomy with or without intravenous thrombolysis in the treatment of acute basilar artery occlusion ischemic stroke: an updated systematic review and meta-analysis

**DOI:** 10.3389/fneur.2024.1433158

**Published:** 2024-10-24

**Authors:** Shuyi Tian, Mengqing Zou, Dan Li, Hang Zhou, Chenghan Wang, Qianshuo Liu, Lianbo Gao

**Affiliations:** The Fourth Clinical College of China Medical University, Shenyang Liaoning, China

**Keywords:** thrombectomy, intravenous thrombolysis, basilar artery, stroke, endovascular therapy (EVT)

## Abstract

**Background:**

Mechanical thrombectomy (MT) is a well-established treatment for acute basilar artery occlusion (BAO)-induced posterior circulation ischemic stroke.

**Objective:**

The objective of the study was to compare the outcomes of endovascular therapy (EVT) with and without bridging intravenous thrombolysis (IVT) in patients with acute BAO, using an updated meta-analysis.

**Methods:**

A systematic literature search was conducted to identify studies that compared the efficacy and safety of EVT with and without IVT in the treatment of acute BAO ischemic stroke. The extracted data included sample size, patient age, National Institutes of Health Stroke Scale (NIHSS) scores, 90-day modified Rankin Scale (mRS) scores of 0–2 and 0–3, mortality rates, symptomatic intracranial hemorrhage (sICH), and occurrence of subarachnoid hemorrhage (SAH).

**Results:**

Five studies that included a total of 1,578 patients (594 IVT + EVT vs. 984 EVT), met the inclusion criteria and were analyzed. The meta-analysis demonstrated that bridging IVT was associated with a higher likelihood of achieving a 90-day mRS score of 0–2 (41% vs. 34%; OR = 1.35, 95% CI 1.09–1.68, *p* = 0.006). Furthermore, the mortality rate was significantly lower in the IVT + EVT group than in the direct EVT group (25% vs. 30%; OR = 0.70, 95% CI 0.55–0.89, *p* = 0.003), with low heterogeneity observed (*I*^2^ = 0.0%, *p* = 0.78). However, there were no significant differences between the groups regarding the rates of sICH (5% vs. 6%; OR = 0.85, 95% CI: 0.52–1.39, *p* = 0.53), SAH (3% vs. 3%; OR = 0.93, 95% CI: 0.39–2.22, *p* = 0.87), perforation (2% vs. 3%; OR = 0.71, 95% CI 0.26–1.95, *p* = 0.51), and dissection (3% vs. 2%; OR = 0.97, 95% CI: 0.13–7.14, *p* = 0.98).

**Conclusion:**

Bridging IVT in conjunction with EVT was associated with better functional outcomes and reduced mortality rates in patients with acute ischemic stroke (AIS) due to BAO compared to EVT alone, without an increased risk of sICH, SAH, perforation, and dissection. In addition, the benefit of bridging IVT to EVT appeared to be more pronounced in European patients than in Asian patients compared to EVT alone. However, the conclusions of this study are not definitive and require validation through large-scale randomized controlled trials (RCTs) to draw more robust conclusions.

**Systematic review registration:**

https://www.crd.york.ac.uk/prospero/, identifier CRD42024531363.

## 1 Introduction

Acute BAO-induced stroke is one of the most severe neurological conditions, accounting for 1%–4% of all acute ischemic strokes, and is characterized by high rates of disability and mortality (1–3). The reported mortality rate for patients with untreated or non-intervened BAO ranges from 80% to 95% (4). Rapid diagnosis and restoration of blood flow are crucial for a favorable prognosis in patients with BAO. Early vascular recanalization and salvage of the ischemic penumbra are key components in the treatment of BAO (5).

Several studies have demonstrated the safety and efficacy of MT for treating posterior circulation ischemic stroke caused by acute BAO (6). Recent RCTs have provided clarity on the treatment of patients with BAO-induced acute ischemic stroke (AIS), demonstrating that EVT combined with optimal pharmacotherapy, including IVT, is superior to optimal pharmacotherapy alone in improving functional outcomes within 24 h of symptom onset (7, 8). Thrombolytics facilitate clot dissolution or dislodgement, thereby increasing clot retrieval and recanalization success rates (9). However, thrombolytic therapy may elevate the risk of bleeding and complicate EVT by rendering clots more difficult to access, potentially complicating the surgical procedure (10). The efficacy and safety of administering thrombolytic agents prior to thrombectomy in patients with acute ischemic stroke remain uncertain (11).

Currently, no RCTs have directly compared the outcomes of bridging IVT prior to EVT vs. EVT alone in patients with acute BAO. Guo et al. (12) found no significant differences in 90-day functional outcome, the likelihood of sICH, or mortality rates between patients receiving bridging IVT before EVT and those receiving direct EVT. More recently, Maïer et al. (13) conducted a propensity score matching analysis, concluding that EVT yielded similar neurological outcomes to IVT combined with EVT, with a comparable safety profile. However, these studies were limited by small sample sizes and patient heterogeneity. Cai et al. (14) conducted a meta-analysis comparing endovascular thrombectomy with and without intravenous thrombolysis in patients with acute BAO, but this study included heterogeneous populations and lacked strict inclusion criteria. Consequently, we conducted an updated meta-analysis with more stringent literature screening and well-defined inclusion criteria to compare the outcomes of direct EVT vs. IVT combined with EVT.

In 2022, Lee et al. (15) published a meta-analysis comparing the clinical outcomes of MT with and without bridging IVT in patients with acute BAO. This meta-analysis indicated that bridging IVT was associated with lower 90-day mortality rates compared to direct MT, particularly in patients with large artery atherosclerosis (LAA), who were more likely to benefit from bridging IVT in terms of better functional outcomes. Subsequently, two new studies on the same topic were published in 2023 (12, 13). However, the conclusions of these studies differed from those of Lee’s meta-analysis. Therefore, we present a pooled analysis and evidence update comparing the clinical outcomes of MT with and without bridging IVT in patients with acute BAO.

## 2 Methods

This evidence-based analysis followed the PRISMA 2020 statement (16). This study was registered on PROSPERO (registration number: CRD42024531363).

### 2.1 Literature search

A systematic literature search was conducted using PubMed, Embase, Cochrane, and Web of Science to identify English-language studies published through February 2024 that compared the efficacy and safety of thrombectomy with or without intravenous thrombolysis in the treatment of acute ischemic stroke due to basilar artery occlusion. The search included both synonyms and related terms, using the following keywords: “thrombectomy,” “intravenous thrombolysis,” “basilar artery,” and “stroke.” The detailed search strategy is provided in Supplementary Table 1.

### 2.2 Eligibility criteria

Studies were included if they met the following criteria: (1) they involved adult patients who experienced an acute BAO ischemic stroke; (2) they evaluated the efficacy and safety of EVT with or without IVT, with IVT administered within 4.5 h according to standard criteria for eligible patients; (3) they reported at least one of the following outcomes: a mRS score of 0–2 at 90 days, mortality, sICH, a mRS score of 0–3 at 90 days, or SAH; (4) they used a primary study design that was either interventional or observational. Reviews, meta-analyses, case reports, conference abstracts, pediatric articles, unpublished articles, replies, editorial comments, studies without sufficient data, and unrelated topics were excluded from the analysis. Non-English language articles were also excluded.

### 2.3 Data extraction

Data extraction was independently performed by two investigators (ST and MZ), with any disagreements resolved by a third investigator (LG) to reach a final decision. To ensure standardization and consistency, data were extracted according to a predefined *pro forma* that included the following variables: first author, publication year, study period, region, study design, sample size, age, male, follow-up, hypertension, admission NIHSS, onset to puncture (OTP), mRS scores of 0–2 at 90 days, mortality, sICH, mRS scores of 0–3 at 90 days, and SAH. In cases of discrepancies or missing data, the corresponding authors were contacted to obtain complete information.

### 2.4 Quality assessment

The quality of the included studies was assessed using the Newcastle–Ottawa Scale (NOS). A score of 7–9 on this scale was considered indicative of high-quality studies. Two investigators (ST and DL) independently evaluated the quality and evidence level of the eligible studies, resolving any disagreements by discussion. Full details of the assessment are provided in Supplementary Table 2.

### 2.5 Statistical analysis

This study used Review Manager 5.4.1 to perform statistical analyses, using OR values to compare binary variables. All results were reported with 95% confidence intervals (CIs). Study heterogeneity was assessed using the chi-squared (χ^2^) test (Cochran’s Q) and the inconsistency index (I^2^) (17). A *p* value of <0.05 or an *I*^2^ > 50% indicated significant heterogeneity. A random-effects model evaluated OR for significant heterogeneity (*p* value <0.05 or *I*^2^ > 50%); otherwise, a fixed-effects model was used for non-significant heterogeneity. Moreover, one-way sensitivity analyses were conducted to evaluate the impact of individual studies on the overall results for outcomes showing significant heterogeneity. Publication bias was assessed both visually and quantitatively using funnel plots generated in Review Manager 5.4.1, where significant asymmetry suggested potential bias. Egger’s regression test was also performed to quantitatively analyze funnel plot asymmetry, which shows statistically significant publication bias with a *p* value of <0.05.

### 2.6 Subgroup design

We also used Review Manager 5.4.1 to perform subgroup analyses based on the study design and geographical region of the included studies. The 95% confidence intervals (CIs) were calculated for four primary outcomes: mortality, mRS scores of 0–2 at 90 days, sICH, and modified treatment in cerebral ischemia (mTICI) grades 2b-3. Study heterogeneity was assessed using the *p* value and inconsistency index (*I*^2^). A *p* value of less than 0.05 or an *I*^2^ greater than 50% indicated significant heterogeneity.

## 3 Results

### 3.1 Literature search and patient baseline characteristics

**Figure 1:**
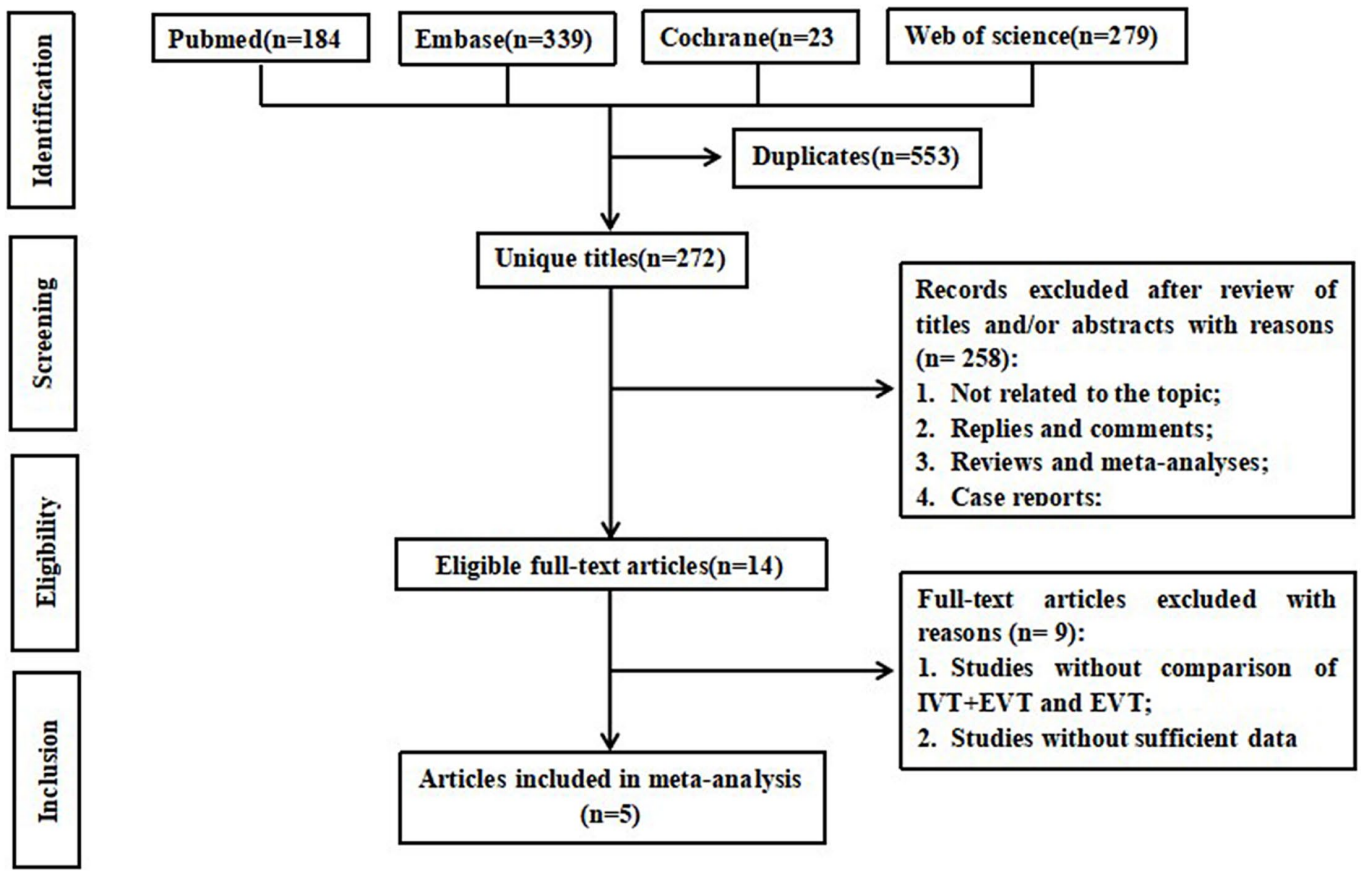
Flowchart of the systematic search and selection process.

Figure 1 illustrates the flowchart of the literature search and selection process. A systematic literature search yielded 825 relevant articles from PubMed (*n* = 184), Embase (*n* = 339), Cochrane (*n* = 23), and Web of Science (*n* = 279). After removing duplicates, 272 articles remained for further review. Of these, 14 publications underwent a full-text review, and ultimately, five studies (12, 13, 18–20) that compared EVT bridging IVT with direct EVT in 1578 BAO stroke patients (594 EVT + IVT vs. 984 EVT) met the inclusion criteria for our meta-analysis. Of these, three (13, 19, 20) were prospective cohort studies, while two (12, 18) were retrospective cohort studies. The studies included in the analysis ranged from 2011 to 2021. Data were collected across large-volume neuro-intervention centers in seven countries: China, Germany, Sweden, Singapore, Italy, France, and the United Kingdom. In the IVT + EVT group, the number of patients included in the EVT + IVT group ranged from 69 to 166, while in the EVT group, it ranged from 118 to 298. The estimated age of the patients was between 60 and 70 years. Baseline data did not significantly differ between the two groups. Further details of the included studies are provided in Table 1. The quality of the eligible studies was assessed using the NOS (Supplementary Table 2). All patients were followed up for 90 days.

**Table 1:** Baseline characteristics of the included studies.

Author + year	Study period	Region	Study design	Patients (*n*)	Follow-up (days)	Age (years)	Male patients (%)	Hypertension (%)	NIHSS on admission
				IVT + EVT/EVT		IVT + EVT/EVT	IVT + EVT/EVT	IVT + EVT/EVT	IVT + EVT/EVT
Maïer 2023	2015–2021	France	Prospective	114/132	90	66.7 ± 16.0/66.8 ± 15.0	67.5/63.6	55.3/63.6	14 ± 14/13 ± 11
Guo 2023	2014–2019	China	Retrospective	118/118	90	62.0 (56.0–71.0)/63.0 (54.0–71.0)	74.6/74.6	70.3/70.3	28 (18–34)/27 (21–33)
Siow 2022	2015–2019	Five countries: Germany/United Kingdom/Singapore/Taiwan/Sweden	Retrospective	127/195	90	69.4 ± 14.0/66.3 ± 14.0	57.5/68.2	83.0/71.4	14 (8–22)/17 (8–26)
Nie 2021	2018–2020	China	Prospective	69/241	90	60.54 ± 9.11/61.63 ± 11.40	78.3/77.2	72.5/69.3	20 (9–27)/21 (12–27)
Nappini 2021	2011–2017	Italian	Prospective	166/298	90	67.1 ± 13.42/67.9 ± 13.22	64.5/65.8	72.9/65.1	NR/NR

### 3.2 Good functional outcome: mRS score of 0–2 at 90 days

**Figure 2:**
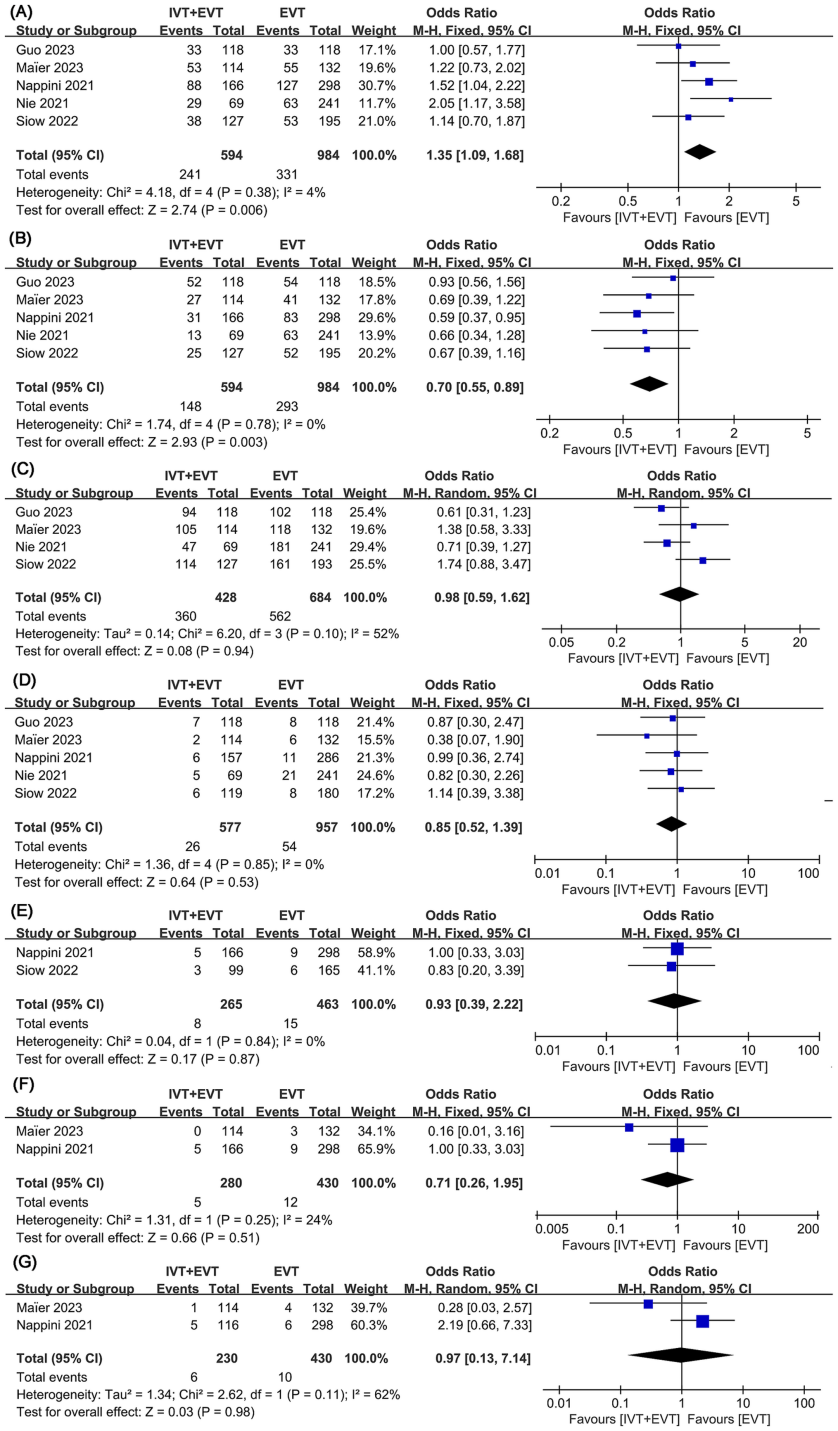
Forest plots of perioperative outcomes. **(A)** mRS 0–2 at 90 days, **(B)** mortality, **(C)** mTICI 2b-3, **(D)** symptomatic intracranial hemorrhage (sICH), **(E)** subarachnoid hemorrhage (SAH), **(F)** perforations, and **(G)** dissections.

**Figure 3:**
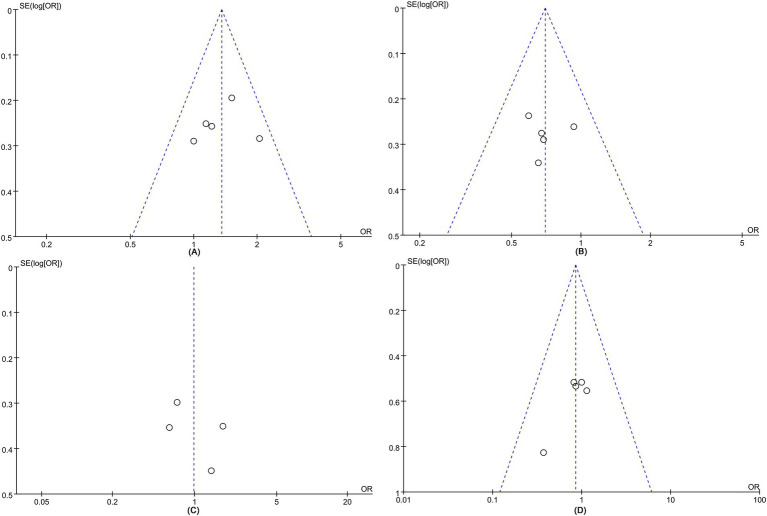
Funnel plots of **(A)** mRS 0–2 at 90 days, **(B)** mortality, **(C)** mTICI 2b-3, and **(D)** symptomatic intracranial hemorrhage (sICH).

Data on the mRS score of 0–2 at 90 days were synthesized from five studies comprising a total of 1,578 patients (594 IVT + EVT vs. 984 EVT) (12, 13, 18–20). A pooled analysis demonstrated that bridging IVT significantly improved the 90-day mRS scores of 0–2 (IVT + EVT 241/594 [41%] vs. EVT 331/984 [34%]; OR = 1.35, 95% CI: 1.09–1.68, *p* = 0.006) (Figure 2A). A visual assessment using a funnel plot suggested a slight publication bias (Figure 3A). However, Egger’s test for publication bias was not statistically significant (*p* = 0.722).

### 3.3 Mortality

Mortality analysis was performed in five studies involving a total of 1,578 patients (594 IVT + EVT vs. 984 EVT) (12, 13, 18–20). The pooled analysis indicated a significantly higher mortality in the direct EVT group (IVT + EVT 148/594 [25%] vs. EVT 293/984 [30%]; OR = 0.70, 95% CI 0.55–0.89, *p* = 0.003) with low heterogeneity (*I*^2^ = 0.0%, *p* = 0.78) (Figure 2B). Funnel plots revealed a slight publication bias (Figure 3B), while Egger’s test detected no statistically significant publication bias (*p* = 0.952).

### 3.4 Successful revascularization: mTICI 2b-3

Four studies involving 1,112 patients (428 IVT + EVT vs. 684 EVT) were included in the analysis (12, 13, 18, 19). Pooled results demonstrated similar mTICI 2b-3 rates between bridging IVT to EVT and direct EVT groups (IVT + EVT 360/428 [84%] vs. EVT 562/684 [82%]; OR = 0.98, 95% CI 0.59–1.62, *p* = 0.94), with no significant heterogeneity (*I*^2^ = 52%, *p* = 0.10) (Figure 2C). No statistically significant publication bias was detected by Egger’s test (*p* = 0.498), nor was any visually evident in the funnel plot (Figure 3C).

### 3.5 Safety outcomes

This analysis found no significant difference in safety results between the two groups. Five studies on sICH involving 1,534 patients (577 IVT + EVT vs. 957 EVT) were included in the analysis (12, 13, 18–20). The evidence showed similar sICH rates between the two groups (IVT + EVT 26/577 [5%] vs. EVT 54/957 [6%]; OR = 0.85, 95% CI 0.52–1.39, *p* = 0.53) without significant heterogeneity (*I*^2^ = 0%, *p* = 0.53) (Figure 2D) or statistically (Egger’s test, *p* = 0.064) or visually (Figure 3D) evident publication bias. Two studies on SAH involving 728 patients (265 IVT + EVT vs. 463 EVT) were included in the analysis (18, 20). Two studies on perforations involving 596 patients (166 IVT + EVT vs. 430 EVT) were included in the analysis (13, 20). Two studies on dissections involving 740 patients (310 IVT + EVT vs. 430 EVT) were included in the analysis (13, 20). Rates of SAH (IVT + EVT 8/265 [3%] vs. EVT 15/463 [3%]; OR = 0.93, 95% CI 0.39–2.22, *p* = 0.87) (Figure 2E), perforations (IVT + EVT 5/280 [2%] vs. EVT 12/430 [3%]; OR = 0.71, 95% CI 0.26–1.95, *p* = 0.51) (Figure 2F), and dissections (IVT + EVT 6/230 [3%] vs. EVT 10/430 [2%]; OR = 0.97, 95% CI 0.13–7.14, *p* = 0.98) (Figure 2G) did not significantly differ between the two groups.

### 3.6 Sensitivity analyses

**Figure 4:**
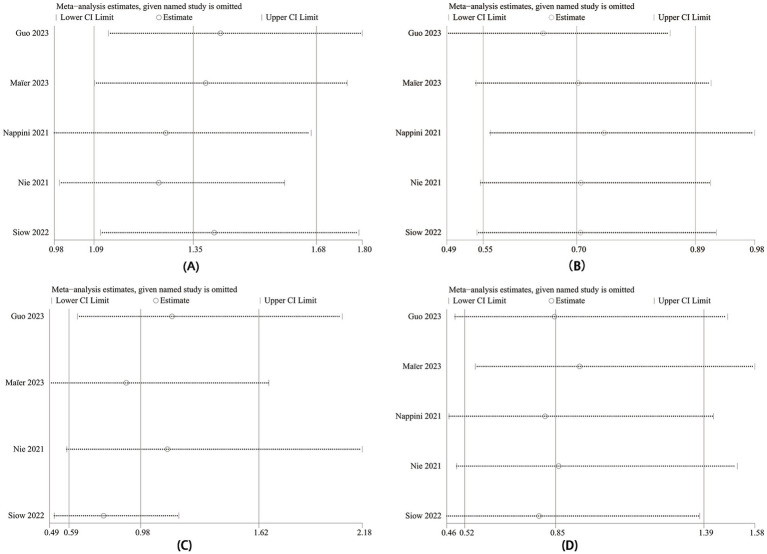
Sensitivity analysis of **(A)** mRS 0–2 at 90 days, **(B)** mortality, **(C)** mTICI 2b-3, and **(D)** sICH.

We conducted single sensitivity analyses for mortality, 90-day mRS score 0–2, mTICI 2b-3, and sICH to evaluate each individual study’s influence on the combined OR through one-by-one exclusion. Sensitivity analyses revealed constant combined ORs after excluding any individual study for mortality (Figure 4B), mTICI 2b-3 (Figure 4C), and sICH (Figure 4D). However, excluding the data from Nappini et al. (20) (95% CI 0.98–1.66) and Nie et al. (19) (95% CI 0.99–1.59) rendered the 90-day mRS 0–2 difference (Figure 4A) insignificant from significant. Consequently, insufficient evidence supports bridging IVT therapy providing better functional outcomes than direct EVT therapy.

### 3.7 Subgroup analysis

Regarding the design and region of the included studies, we conducted the subgroup analysis in Table 2. All three prospective studies confirmed that bridging IVT prior to EVT was associated with better functional outcomes and lower mortality than direct EVT in acute basilar artery occlusion ischemic stroke patients, which is consistent with our overall analysis. A pooled subgroup analysis of European patients confirmed that 90-day mortality rates favored bridging IVT to EVT [OR = 0.63 (95% CI 0.44–0.90); *p* < 0.05], and bridging IVT was associated with better functional outcomes [OR = 1.40 (95% CI 1.03–1.90); *p* < 0.05]. However, no significant difference existed between the two groups in the Asian population.

**Table 2:** Subgroup analysis of IVT + EVT vs. EVT for acute basilar artery occlusion ischemic stroke.

Subgroup	Mortality				mRS 0–2 at 90 days				sICH				mTICI 2b-3			
	Study	OR [95%CI]	*p* value	*I*^2^	Study	OR [95%CI]	*p* value	*I*^2^	Study	OR [95%CI]	*p* value	*I*^2^	Study	OR [95%CI]	*p* value	*I*^2^
Total	5	0.70 [0.55–0.89]	0.003	0%	5	1.35 [1.09–1.68]	0.006	4%	5	0.85 [0.52–1.39]	0.53	0%	4	0.98 [0.59–1.62]	0.94	52%
Study design																
Prospective	3	0.64 [0.46–0.87]	0.005	0%	3	1.52 [1.17–1.99]	0.002	0%	3	0.77 [0.40–1.47]	0.43	0%	2	0.91 [0.48–1.72]	0.77	36%
Retrospective	2	0.80 [0.55–1.16]	0.23	0%	2	1.08 [0.74–1.57]	0.69	0%	2	0.99 [0.47–2.10]	0.98	0%	2	1.04 [0.37–2.88]	0.95	77%
Region																
Asia	2	0.81 [0.54–1.22]	0.32	0%	2	1.43 [0.96–2.12]	0.08	68%	2	0.84 [0.41–1.74]	0.64	0%	2	0.67 [0.43–1.04]	0.08	0%
Europe	2	0.63 [0.44–0.90]	0.01	0%	2	1.40 [1.03–1.90]	0.03	0%	2	0.73 [0.31–1.71]	0.47	0%	1	1.38 [0.58–3.33]	0.47	NA

## 4 Discussion

Acute BAO is a rare but potentially catastrophic condition. Earlier studies have demonstrated that early recanalization of occluded arteries in acute stroke is associated with a good functional prognosis (21). Currently, revascularization therapy forms include IVT, arterial thrombolysis, MT, stent implantation, or combination therapy (22). EVT is a well-established approach for acute ischemic stroke. Several studies have analyzed conflicting results on the efficacy of bridging IVT vs. direct MT; some have reported similar results (23–25), while other studies have shown that bridging IVT therapy has a better functional outcome (26). However, the majority of the analyses in these articles were based on anterior circulation strokes, not specifically studying acute BAO. The benefit of bridging IVT before EVT in BAO patients remains uncertain, and the optimal clinical treatment for acute BAO requires clarification.

This study found that bridging IVT prior to EVT improved 90-day functional outcomes and reduced mortality in BAO patients. No significant difference existed in vascular revascularization rate or safety prognosis between the two groups. However, this result may be limited by the sample size, resulting in type II errors. Sensitivity analysis indicated that when the data of Nappini et al. (20) and Nie et al. (19) were excluded, the heterogeneity for 90-day mRS score 0–2 disappeared, suggesting that these studies accounted for the majority of the heterogeneity. This result may be due to the large number of patients in the study by Nappini et al. (20). At present, there are few studies on endovascular therapy (EVT) with and without bridging intravenous thrombolysis (IVT) in acute BAO. Only five studies were included in this meta-analysis after our rigorous screening. It is expected that more new RCTs will appear in the future to solve this heterogeneity problem. Moreover, among all included studies, Egger’s test was not statistically significant.

In our subgroup analysis, prospective studies confirmed that bridging IVT prior to EVT was associated with better functional outcomes and lower mortality than direct EVT in patients, which is consistent with our overall analysis. Prospective studies are generally more accurate than retrospective studies due to the inherent limitations of the retrospective and observational nature of the included studies. Furthermore, we found that European patients benefited more from bridging IVT than Asian patients. Relevant epidemiological studies have shown that intracranial atherosclerotic disease (ICAD) accounts for 10–15% of ischemic strokes in European patients, but ICAD causes 54% of ischemic strokes in Asian populations (27, 28).

Some studies have demonstrated that there are racial differences in the prevalence, severity, site of onset, pathogenesis, and prognosis of ICAD. These differences may be attributed, at least in part, to the configuration of risk factors that vary between groups (29–31). Pathophysiological studies have shown that atherosclerosis is the deposition of cholesterol in the arterial wall to form atherosclerotic plaques. A simplified model of the pathogenesis of atherosclerosis begins with endothelial dysfunction and the accumulation of cholesterol particles in the arterial wall (32, 33). Shear stress affects the function of endothelial cells, and the hemodynamic environment, which is normally characterized by low wall shear and peripheral wall tension, promotes the development of atherosclerosis. Wall tension serves to compensate for non-laminar flow patterns (34). As arteries stiffen from prolonged compression, especially in hypertensive patients, the wall tension that compensates for non-laminar flow patterns may become fatigued. This may contribute to the development of intracranial atherosclerotic disease as hypertension, smoking, and obesity—which are risk factors that may mediate endothelial cell dysfunction by affecting shear forces—are more prevalent in Asian populations (30, 34). These risk factors may potentially mediate endothelial cell dysfunction by affecting shear forces, which could result in variations in disease development among patients of different ethnic groups (29, 30). In this study population, the differences in treatment outcomes between the two groups may also be due to ethnicity and genetic factors, as along with other factors such as underlying disease, risk factors, and lifestyle (other influencing factors not included in the baseline comparison). However, there is currently no clear evidence to suggest that European subjects are more suitable candidates for bridging therapy than Asian subjects for BAO treatment.

In recent years, three high-quality studies on BAO treatment in China—ATTENTION, BAOCHE, and BEST trials, and one European study—BASICS trial (7, 8, 35, 36), have been published. Comparative analysis revealed lower IVT usage before EVT in the ATTENTION (31%), BAOCHE (14%), and BEST (27%) trial EVT groups compared to the BASICS trial (78.6%) (7, 8, 35, 36). However, the 90-day mRS 0–2 functional independence rate in the EVT group remained numerically similar between the ATTENTION, BAOCHE, and BASICS trials. Due to differences in sample size, among other factors, this analysis cannot explain the discrepancy between the good prognosis in the BASICS trial and the similarity observed in the Chinese trials.

Studies have shown that direct EVT is no less effective than bridging therapy within 4.5 h of onset in patients with acute ischemic stroke (AIS) combined with precirculatory large-vessel occlusion (LVO) (37). However, few studies have compared IVT + EVT and direct EVT in patients with acute posterior circulation BAO. Bridging EVT therapy after IVT in LVO offers potential advantages, including early thrombus fragmentation, microvascular reperfusion, and enhanced recanalization (37–39). Posterior circulation occlusion is significantly associated with distal embolization during thrombectomy, where bridging tissue plasminogen activators may partially ameliorate this situation, resulting in better outcomes (40, 41).

Nappini et al. (20) showed that patients with direct EVT and IVT + EVT had similar rates of recurrence, symptomatic intracranial hemorrhage, and 3-month functional outcomes, but patients treated with IVT + EVT within the first 6 h had a better prognosis. More recently, Siow et al. (18) also found similar good mobility rates (3-month mRS 0–3), symptomatic intracranial hemorrhage, and mortality in patients treated with EVT alone and IVT + EVT. Consistent with this meta-analysis result, bridging IVT before EVT has potential risks and benefits. Currently, no exact evidence-based medicine or standardized clinical guidelines prove IVT + EVT’s overwhelming advantage over direct EVT in acute posterior circulation BAO. Individualized treatment options for patients with acute BAO need exploration, and larger randomized studies are needed to provide more evidence.

The included studies in this meta-analysis had inherent limitations due to their retrospective and observational nature. Given the included studies’ observational nature, the decision to perform IVT before EVT was not random, resulting in significant allocation bias and residual unmeasured confounders that may have affected the results. In addition, only five studies were included after a rigorous systematic literature search, providing an overall small sample size and potentially insufficient statistical power to detect significant efficacy results. Large-scale, precise, prospective randomized studies with long-term follow-up are needed to further compare the two therapeutic approaches’ good functional outcomes, mortality, and complications in patients with acute BAO.

## 5 Conclusion

This meta-analysis found that bridging IVT was associated with better functional outcomes and lower mortality rates in patients with BAO-induced AIS compared to direct EVT, without increasing rates of sICH, SAH, perforation, and dissection. European patients benefited more from bridging IVT to EVT than Asian patients did from EVT alone. However, due to heterogeneity and potential bias, the conclusion of this study is not stable, and the level of evidence is low. Neurologists should choose the most appropriate treatment according to the patient’s specific situation. Large randomized clinical trials are needed to evaluate whether bridging IVT indeed provides more benefits over direct EVT in BAO.

## Data Availability

The original contributions presented in the study are included in the article/Supplementary material, further inquiries can be directed to the corresponding author.
