# Supplementary material for: Efficacy and safety of thrombectomy with or without intravenous thrombolysis in the treatment of acute basilar artery occlusion ischemic stroke: an updated systematic review and meta-analysis
Source: Front Neurol. 2024 Oct 24;15:1433158. doi: 10.3389/fneur.2024.1433158 (PMC11540773; doi:10.3389/fneur.2024.1433158)
Supplement: Supplementary file 1 [file Table_1.DOCX]

Supplementary Material

| **Supplementary Table 1** Search strategy | | |
| --- | --- | --- |
| **Database** | **search strategy** | **Number** |
| **PubMed** | **(((("Thrombectomy"[Mesh]) OR (((((((((((Thrombectomies) OR (Percutaneous Aspiration Thrombectomy)) OR (Aspiration Thrombectomies, Percutaneous)) OR (Aspiration Thrombectomy, Percutaneous)) OR (Percutaneous Aspiration Thrombectomies)) OR (Thrombectomies, Percutaneous Aspiration)) OR (Thrombectomy, Percutaneous Aspiration)) OR (Aspiration Thrombectomy)) OR (Aspiration Thrombectomies)) OR (Thrombectomies, Aspiration)) OR (Thrombectomy, Aspiration))) AND (("Thrombolytic Therapy"[Mesh]) OR (((((((((((((Therapeutic Thrombolysis) OR (Therapeutic Thrombolyses)) OR (Thrombolyses, Therapeutic)) OR (Thrombolysis, Therapeutic)) OR (Therapy, Fibrinolytic)) OR (Fibrinolytic Therapies)) OR (Therapies, Fibrinolytic)) OR (Therapy, Thrombolytic)) OR (Therapies, Thrombolytic)) OR (Thrombolytic Therapies)) OR (Fibrinolytic Therapy)) OR (intravenous thrombolysis)) OR (IVT)))) AND (("Basilar Artery"[Mesh]) OR ((((Arteries, Basilar) OR (Artery, Basilar)) OR (Basilar Arteries)) OR (basilar artery occlusion)))) AND (("Stroke"[Mesh]) OR ((((((((((((((((((((((((((((Strokes) OR (Cerebrovascular Accident)) OR (Cerebrovascular Accidents)) OR (CVA (Cerebrovascular Accident))) OR (CVAs (Cerebrovascular Accident))) OR (Cerebrovascular Apoplexy)) OR (Apoplexy, Cerebrovascular)) OR (Vascular Accident, Brain)) OR (Brain Vascular Accident)) OR (Brain Vascular Accidents)) OR (Vascular Accidents, Brain)) OR (Cerebrovascular Stroke)) OR (Cerebrovascular Strokes)) OR (Stroke, Cerebrovascular)) OR (Strokes, Cerebrovascular)) OR (Apoplexy)) OR (Cerebral Stroke)) OR (Cerebral Strokes)) OR (Stroke, Cerebral)) OR (Strokes, Cerebral)) OR (Stroke, Acute)) OR (Acute Stroke)) OR (Acute Strokes)) OR (Strokes, Acute)) OR (Cerebrovascular Accident, Acute)) OR (Acute Cerebrovascular Accident)) OR (Acute Cerebrovascular Accidents)) OR (Cerebrovascular Accidents, Acute)))** | **184** |
| **EMBASE** | **((Thrombectomy or (Thrombectomies or Percutaneous Aspiration Thrombectomy or Aspiration Thrombectomies,Percutaneous orAspiration Thrombectomy,Percutaneous or Percutaneous Aspiration Thrombectomies or Thrombectomies,Percutaneous Aspiration orThrombectomy,Percutaneous Aspiration or Aspiration Thrombectomy or Aspiration Thrombectomies or Thrombectomies,Aspiration orThrombectomy,Aspiration))and (Thrombolytic Therapy or (Therapeutic Thrombolysis or Therapeutic Thrombolyses or Thrombolyses,Therapeutic or Thrombolysis,Therapeutic or Therapy,Fibrinolytic or Fibrinolytic Therapies or Therapies,Fibrinolytic or Therapy,Thrombolytic or Therapies,Thrombolytic or Thrombolytic Therapies or Fibrinolytic Therapy or intravenous thrombolysis or IVT))and(Basilar Artery or (Arteries,Basilar or Artery,Basilar or Basilar Arteries or basilar artery occlusion))and (Stroke or (Strokes orCerebrovascular Accident or Cerebrovascular Accidents or CVA or CVAs or Cerebrovascular Apoplexy or Apoplexy,Cerebrovascular orVascular Accident,Brain or Brain Vascular Accident or Brain Vascular Accidents or Vascular Accidents,Brain or Cerebrovascular Stroke orCerebrovascular Strokes or Stroke,Cerebrovascular or Strokes,Cerebrovascular or Apoplexy or Cerebral Stroke or Cerebral Strokes orStroke,Cerebral or Strokes,Cerebral or Stroke,Acute or Acute Stroke or Acute Strokes or Strokes,Acute or Cerebrovascular Accident,Acute or Acute Cerebrovascular Accident or Acute Cerebrovascular Accidents or Cerebrovascular Accidents,Acute))).af.** | **339** |
| **Cochrane** | **((Thrombectomy or (Thrombectomies or Percutaneous Aspiration Thrombectomy or Aspiration Thrombectomies,Percutaneous orAspiration Thrombectomy,Percutaneous or Percutaneous Aspiration Thrombectomies or Thrombectomies,Percutaneous Aspiration orThrombectomy,Percutaneous Aspiration or Aspiration Thrombectomy or Aspiration Thrombectomies or Thrombectomies,Aspiration orThrombectomy,Aspiration))and (Thrombolytic Therapy or (Therapeutic Thrombolysis or Therapeutic Thrombolyses or Thrombolyses,Therapeutic or Thrombolysis,Therapeutic or Therapy,Fibrinolytic or Fibrinolytic Therapies or Therapies,Fibrinolytic or Therapy,Thrombolytic or Therapies,Thrombolytic or Thrombolytic Therapies or Fibrinolytic Therapy or intravenous thrombolysis or IVT))and(Basilar Artery or (Arteries,Basilar or Artery,Basilar or Basilar Arteries or basilar artery occlusion))and (Stroke or (Strokes orCerebrovascular Accident or Cerebrovascular Accidents or CVA or CVAs or Cerebrovascular Apoplexy or Apoplexy,Cerebrovascular orVascular Accident,Brain or Brain Vascular Accident or Brain Vascular Accidents or Vascular Accidents,Brain or Cerebrovascular Stroke orCerebrovascular Strokes or Stroke,Cerebrovascular or Strokes,Cerebrovascular or Apoplexy or Cerebral Stroke or Cerebral Strokes orStroke,Cerebral or Strokes,Cerebral or Stroke,Acute or Acute Stroke or Acute Strokes or Strokes,Acute or Cerebrovascular Accident,Acute or Acute Cerebrovascular Accident or Acute Cerebrovascular Accidents or Cerebrovascular Accidents,Acute))).af.** | **23** |
| **Web of Science** | **((Thrombectomy or (Thrombectomies or Percutaneous Aspiration Thrombectomy or Aspiration Thrombectomies,Percutaneous orAspiration Thrombectomy,Percutaneous or Percutaneous Aspiration Thrombectomies or Thrombectomies,Percutaneous Aspiration orThrombectomy,Percutaneous Aspiration or Aspiration Thrombectomy or Aspiration Thrombectomies or Thrombectomies,Aspiration orThrombectomy,Aspiration))and (Thrombolytic Therapy or (Therapeutic Thrombolysis or Therapeutic Thrombolyses or Thrombolyses,Therapeutic or Thrombolysis,Therapeutic or Therapy,Fibrinolytic or Fibrinolytic Therapies or Therapies,Fibrinolytic or Therapy,Thrombolytic or Therapies,Thrombolytic or Thrombolytic Therapies or Fibrinolytic Therapy or intravenous thrombolysis or IVT))and(Basilar Artery or (Arteries,Basilar or Artery,Basilar or Basilar Arteries or basilar artery occlusion))and (Stroke or (Strokes orCerebrovascular Accident or Cerebrovascular Accidents or CVA or CVAs or Cerebrovascular Apoplexy or Apoplexy,Cerebrovascular orVascular Accident,Brain or Brain Vascular Accident or Brain Vascular Accidents or Vascular Accidents,Brain or Cerebrovascular Stroke orCerebrovascular Strokes or Stroke,Cerebrovascular or Strokes,Cerebrovascular or Apoplexy or Cerebral Stroke or Cerebral Strokes orStroke,Cerebral or Strokes,Cerebral or Stroke,Acute or Acute Stroke or Acute Strokes or Strokes,Acute or Cerebrovascular Accident,Acute or Acute Cerebrovascular Accident or Acute Cerebrovascular Accidents or Cerebrovascular Accidents,Acute))).af.** | **279** |

| **Supplementary Table 2** Quality evaluation of the eligible studies with Newcastle–Ottawa scale. | | | | | | | | | |
| --- | --- | --- | --- | --- | --- | --- | --- | --- | --- |
| Study | Selection | | | | Comparability | | Outcome | | |
|  | Representative-ness | Selection of  non-exposed | Ascertainment  of exposure | Outcome not present at start | Comparability on most important factors | Comparability on other risk factors | Assessment of outcome | Long enough follow-up (median≥90 days) | Adequacy  (completeness) of follow-up |
| Benjamin 2023 | * | * | * | * | * | * | * | * | * |
| Guo 2023 | * | * | * | * | * | * | * | * | * |
| Isabel 2022 | * | * | * | * | * | - | * | * | * |
| Nie 2022 | * | * | * | * | * | * | * | * | - |
| Sergio 2021 | * | * | * | * | - | - | * | * | * |
| *indicates criterion met; - indicates significant of criterion not met. | | | | | | | | | |
